# A Multi-Omics Approach Using a Mouse Model of Cardiac Malformations for Prioritization of Human Congenital Heart Disease Contributing Genes

**DOI:** 10.3389/fcvm.2021.683074

**Published:** 2021-08-24

**Authors:** Adrianna Matos-Nieves, Sathiyanarayanan Manivannan, Uddalak Majumdar, Kim L. McBride, Peter White, Vidu Garg

**Affiliations:** ^1^Center for Cardiovascular Research and Heart Center, Nationwide Children's Hospital, Columbus, OH, United States; ^2^Department of Pediatrics, Ohio State University, Columbus, OH, United States; ^3^The Institute for Genomic Medicine, Nationwide Children's Hospital, Columbus, OH, United States; ^4^Department of Molecular Genetics, Ohio State University, Columbus, OH, United States

**Keywords:** heart development, congenital heart disease, mouse model, tetralogy of Fallot, human genetics

## Abstract

Congenital heart disease (CHD) is the most common type of birth defect, affecting ~1% of all live births. Malformations of the cardiac outflow tract (OFT) account for ~30% of all CHD and include a range of CHDs from bicuspid aortic valve (BAV) to tetralogy of Fallot (TOF). We hypothesized that transcriptomic profiling of a mouse model of CHD would highlight disease-contributing genes implicated in congenital cardiac malformations in humans. To test this hypothesis, we utilized global transcriptional profiling differences from a mouse model of OFT malformations to prioritize damaging, *de novo* variants identified from exome sequencing datasets from published cohorts of CHD patients. *Notch1*^+/−^*; Nos3*^−/−^ mice display a spectrum of cardiac OFT malformations ranging from BAV, semilunar valve (SLV) stenosis to TOF. Global transcriptional profiling of the E13.5 *Notch1*^+/−^*; Nos3*^−/−^ mutant mouse OFTs and wildtype controls was performed by RNA sequencing (RNA-Seq). Analysis of the RNA-Seq dataset demonstrated genes belonging to the *Hif1α*, *Tgf-β*, *Hippo*, and *Wnt* signaling pathways were differentially expressed in the mutant OFT. Mouse to human comparative analysis was then performed to determine if patients with TOF and SLV stenosis display an increased burden of damaging, genetic variants in gene homologs that were dysregulated in *Notch1*^+/−^*; Nos3*^−/−^ OFT. We found an enrichment of *de novo* variants in the TOF population among the 1,352 significantly differentially expressed genes in *Notch1*^+/−^*; Nos3*^−/−^ mouse OFT but not the SLV population. This association was not significant when comparing only highly expressed genes in the murine OFT to *de novo* variants in the TOF population. These results suggest that transcriptomic datasets generated from the appropriate temporal, anatomic and cellular tissues from murine models of CHD may provide a novel approach for the prioritization of disease-contributing genes in patients with CHD.

## Introduction

As the most common type of birth defect, congenital heart disease (CHD) affects nearly ~1% of all live births (1). Malformations of the cardiac outflow tract (OFT), which include incorrect positioning or septation of the major vessels (aorta and pulmonary artery) as well as anomalies of the aortic or pulmonic (semilunar) valves, account for an estimated 30% of CHD cases (1). These types of malformations range from the simple to the more complex such as bicuspid aortic valve (BAV) and tetralogy of Fallot, respectively. Bicuspid aortic valve (BAV), where the normal trileaflet structure is disrupted and two valve leaflets are instead observed, is the most common type of CHD, with an estimated population prevalence between 1–2% (2). BAV is frequently undiagnosed during infancy since it often does not impact cardiac function at an early age, however afflicted patients are at an increased risk of calcific aortic valve disease (CAVD) and resultant stenosis as adults (3, 4). Tetralogy of Fallot (TOF), is one of the more complex forms of CHD affecting the OFT in which abnormal positioning of the aorticopulmonary septum leads to pulmonic valve stenosis and a ventricular septal defect. TOF requires surgical intervention during infancy, lifelong medical monitoring, and often pulmonic valve replacement as an adult. Other less common cardiac OFT malformations, referred to as conotruncal heart defects, include truncus arteriosus, transposition of the great arteries and double outlet right ventricle. In total, conotruncal heart defects compose a significant and growing portion of adult CHD survivors, but the genetic contributors for the majority of cases have not been defined.

Conotruncal CHD can be traced back to the improper development of the transient yet critical common cardiac OFT (5). The common OFT contributes to the development of the great vessels and the semilunar valves following multiple morphological changes to this initial common structure. In mice, the common OFT is visually distinguishable by embryonic day (E) 9.5 as it is one of four major anatomical components in addition to the common atrium, atrioventricular canal, and common ventricle. At this stage, the OFT is best described as a cylinder of cells of anterior second heart field (SHF) origin that is lined with endothelial cells (6–10). This structure also receives important contributions from migrating cardiac neural crest cells (CNC) (11, 12). Septation of the OFT into the aorta and pulmonary artery is achieved by the migrating population of CNC towardz the SHF and muscularization of this tissue (13, 14). Endocardial cushions, which are the precursors of semilunar valves, form in the outflow region of the primitive heart tube (15). These primitive valve structures are composed of a layer of extracellular matrix that is interposed between the endothelial cells and the surrounding myocardium (15–17). Endothelial cells undergo endothelial to mesenchymal transition (EMT) and populate the endocardial cushions with newly transformed mesenchymal cells (18). This is followed by semilunar valve development and remodeling. Considering that the development of OFT-derived structures is dependent on the migration and differentiation of multiple cell lineages, it is unsurprising that the etiologies of OFT malformations are numerous and complex.

Previous human genetics analyses and mouse gene knockout studies have identified multiple genetic contributors to OFT development and disease. Among these, mutations in *NOTCH1* were among the first implicated to contribute to semilunar valve and OFT malformations following linkage analysis of two kindreds affected with aortic valve disease consisting of BAV, aortic valve stenosis, CAVD along with one individual with TOF (19). A large cohort of patients with a left-ventricular outflow tract malformations were also screened for *NOTCH1* mutations and were found to harbor a significant burden of inherited missense variants (20). Furthermore, a study of 428 probands with familial left-sided CHD demonstrated that those families having members with conotruncal heart disease often had pathogenic variants in *NOTCH1* demonstrating a spectrum of phenotypes associated with *NOTCH1* genetic variation (21). A genome-wide chromosomal analysis of TOF patients identified *de novo* copy number variations in loci known to encode *NOTCH1* and *JAG1*, a ligand for the *NOTCH1* receptor, that were absent in controls further implicating genes in the Notch signaling pathway as potential contributors of disease (22). Exome sequencing methods in TOF patient populations identified disease-contributing variants in *NOTCH1* and also allowed for the characterization of other genes such as *FLT4*, which encodes for VEGFR-3 (23–25). Meanwhile, an inspection of cardiac phenotypes in transgenic mouse models deficient in *Fgf*, *Bmp*, Slit/Robo, *N-Cadherin*, and *Wnt* signaling have also identified malformations of OFT structures with varying degrees of penetrance (6, 26–36).

We have previously published that *Notch1* haploinsufficient mice backcrossed into a *Nos3*-null background are a highly penetrant model of cardiac OFT malformations and semilunar valve disease (37, 38). At E18.5, these mice display a spectrum of phenotypes including thickened, malformed semilunar valves, BAV, and additional anomalies of the OFT including overriding aorta and ventricular septal defect, which are reminiscent of TOF. These cardiac phenotypes were observed in late gestation *Notch1*^+/−^*; Nos3*^−/−^ embryos suggesting that they were the result of abnormal development at earlier timepoints. Deletion of *Notch1* in the endothelial cell and the SHF lineages in this mouse model recapitulated the observed semilunar valve and OFT malformations indicating the importance of *Notch1* in these cells and their derivatives.

Exome and genome sequencing approaches have greatly enhanced the ability to rapidly identify genetic variants in patients with CHD, but the prioritization of the rapidly growing number of variants in regard to pathogenicity has proven to be difficult. Here, we have utilized the gene expression profiling differences identified in a murine model of cardiac OFT malformations to prioritize and strengthen the genetic link between novel gene candidates identified in patients with conotruncal heart disease. First, we performed transcriptomic analysis of dissected OFTs obtained from E13.5 *Notch1*^+/−^*; Nos3*^−/−^ embryonic hearts and identified genes with differential expression patterns when compared to wild-type controls. Single-cell RNA-Sequencing (scRNA-Seq) data generated from wildtype E12.5 cardiac OFTs was used to predict the cell-type specificity of dysregulated genes and genes expressed in non-contributing cell types were excluded. We cross-compared those genetic homologs dysregulated in the *Notch1*^+/−^*; Nos3*^−/−^ mouse OFT to genetic variants identified in published patient cohorts with tetralogy of Fallot (TOF) and semilunar valve (SLV) disease. We identified a significant overlap between genes differentially expressed in the OFT of the *Notch1*^+/−^*; Nos3*^−/−^ mouse model and genes with *de novo* variants in TOF but not SLV patients. Notably, no significant overlap was found when comparing the highest expressing genes in the mouse OFT to the *de novo* variant gene list from TOF patients. Together, this analysis pipeline provides an additional methodology to prioritize disease-causing genetic variants that are likely pathogenic contributors to CHD.

## Materials and Methods

### Experimental Mouse Models

*Notch1* (*Notch1*^+/−^) and endothelial nitric oxide synthase 3 (*Nos3*^−/−^) knockout strains were generated as previously described and are publicly available at the Jackson Laboratory (#002797, and #002684) (39, 40). These mice were housed as live heterozygote colonies to ensure line maintenance and kept in a C57/BL6J background. All mice were maintained on a 12-h-light/dark cycle and fed a standard western diet. For timed breeding (*Notch1*^+/−^*; Nos3*^+/−^ × *Nos3*^+/−^; *Notch1*^+/−^ × wildtype), noon of the day of vaginal plug was observed is defined as embryonic day E0.5. Pregnant dams were anesthetized using inhalation of 3% isoflurane. Cervical dislocation and organ removal were used as a secondary method of euthanasia. Embryos were collected at E12.5 or E13.5 and wildtype littermates were used as controls. All animal experiments were approved by the Institutional Animal Care and Use Committee at the Research Institute at Nationwide Children's Hospital.

### Isolation of RNA From the E13.5 Embryonic Cardiac Outflow Tract and Bulk RNA-Sequencing

E13.5 embryos were collected in an RNase-free environment. A blunt cut at the base of the OFT, below the conal cushions, was used to collect the OFT tissues from each embryo. The OFT were snap frozen until genotyping had been completed using tissue from the remaining embryo. Following genotyping of the embryos, three OFTs were pooled in 1 ml Trizol to form one biological replicate. Three biological replicates of the mutant embryos and three biological replicates of the wild type embryos were used for the final RNA-Seq analysis (i.e., 3 OFTs per sample submitted, 18 total OFTs). Total RNA was collected following tissue homogenization using TissueLyser II (Qiagen) and chloroform-isopropanol extraction and purification and isolated using the Total RNA Purification kit (Norgen Biotek Corp, 17200). RNA-Seq was performed at Ocean Ridge Biosciences as previously described (41). RNA TruSeq Stranded Total RNA LT with Ribo-Zero Gold Set A kit (Illumina, RS-122-2301) was used to generate libraries. Libraries were sequenced using Illumina HiSeq 2500 to generate paired-end 50 bp reads.

### Bioinformatics Analysis of Bulk RNA-Sequencing

The raw FASTQ files were split into files containing 4,000,000 reads and checked for quality using the FASTX-Toolkit1 (version 0.0.14, http://hannonlab.cshl.edu/fastx_toolkit/). The reads were filtered (removing sequences that did not pass Illumina's quality filter) and trimmed based on the quality results (3 nucleotides at the left end of the R1 reads). Then, sequence alignment was performed using TopHat (v2.1.0) to mouse genome version mm10 (ftp://ftp.ccb.jhu.edu/pub/data/bowtie2_indexes/mm10) (42). Following the alignment to the mouse genome, BAM files were merged on a per-sample basis. Generation of BAM files was performed by Ocean Ridge Biosciences. Aligned BAM files are used to count the number of reads mapping to exons in each transcript using the GenomicAlignments (version 1.22.1) package in R to generate a gene-count matrix (43). Differential expression was evaluated from this gene-count matrix using the DESeq2 package (version 1.26.0) using the standard differential expression analysis pipeline (without log fold change shrinkage method) in R (44). Genes with ≤5 reads across all samples were excluded from the analysis. Genes that were differentially expressed were filtered using the cutoff: adjusted *P*-value from DESeq2 result <= 0.05. Genes passing this filtering cutoff were then used as input for a KEGG pathway enrichment analysis using DAVID V6.8 online tool (45, 46). Identified pathways were then classified as signaling pathways, metabolic pathways, and cardiovascular disease-related genes. A chord plot showing gene-pathway relationships in each of these classes was created using a modified GoChord function from the GOplot package in R (47).

### Single-Cell Preparation and Sequencing

E12.5 cardiac OFTs (both *Notch1*^+/−^ and wildtype) were microdissected from five mouse embryos and pooled for single-cell RNA sequencing. To prepare single cells suspensions, pooled OFT tissues were incubated with 1 mg/ml Collagenase II (Worthington Biochemical Corporation# LS004176) for 15 min at 37°C with occasional stirring at every 5 min for complete dissociation. The digestion reaction was quenched immediately with 1 ml. DMEM supplemented with 10% FBS and pelleted down at 1,000 rpm for 5 min at 4°C. Pelleted cells were washed with cold PBS and resuspended in 0.04% BSA in PBS at concentration 9.74 × 10^5^ cells/ml with 83% viability (8.03 × 10^5^ live cells/ml).

A single-cell droplet library of the pooled *Notch1*^+/−^/wildtype OFTs samples was generated using the 10xGenomics Chromium controller from this suspension according to the manufacturer's instructions. The quality and integrity of the cDNA library was quantitated using the High Sensitivity D5000 and D1000 ScreenTape (Agilent# 5067-5592 and 5067-5584) on the Agilent-2200 TapeStation. The library was sequenced (150 bp paired-end) using the Illumina HiSeq 4000 platform at the Steve and Cindy Rasmussen Institute for Genomic Medicine at Nationwide Children's Hospital. A total of 337,081,925 paired end reads and a total of 2,116 cells were detected for the pooled sample. Thus, on average, the transcriptome of each cell was evaluated using 159,301 reads.

### Analysis of scRNA-Seq Data

Illumina.bcl files were demultiplexed and converted into per-sample FASTQ files using the 10x Genomics cellranger “mkfastq” command. The FASTQ files were then used to create a gene-count matrix using the cellranger command “count” using the mm10 genome version index from 10X genomics (https://cf.10xgenomics.com/supp/cell-exp/refdata-gex-mm10-2020-A.tar.gz) modified to include Neomycin resistance sequence using “reform” (https://gencore.bio.nyu.edu/reform/; accessed 09/15/2020). The expression of Neomycin resistance gene (NeoR) was used to identify Notch1 heterozygote cells. The count output file was then imported into the Seurat (v 3.0) package in R (45). Cells with at least 1 read mapping to NeoR gene were removed and the rest of the cells were used for further analysis. These NeoR negative cells were filtered for number of expressed genes and percentage of mitochondrial gene expression, normalized, subject to principal component analysis using the highest variable genes, and scaled as described earlier (46). Following this, dimensional reduction was performed using the RunUMAP and RunTSNE functions of Seurat using the first 20 principal components, and cells were clustered using the Louvain algorithm using a resolution factor of 0.5. The clustered cells were renamed using markers described in literature into vascular smooth muscle cells (VSMC), mesenchymal cells (Mes), endothelial cells (EC), myocardial cells (Myo), epicardial cells (epi), blood and ectodermal cells (48). Blood and ectodermal cells were removed for further analysis and dimensional reduction and clustering was repeated for the mesodermal cells.

Using these clusters, we examined cell-type specific expression of genes. Cell type-specific expression was examined by comparing a gene in an individual cluster against all other cells in the dataset using the “FindAllMarkers” function in R. As changes in the number of cells expressing a gene as well as the changes in the average expression per cell can contribute to differential expression, we used a threshold for both of these attributes. For differential expression between a cluster and rest of the cells, log fold change threshold was set to 0.5 (logfc.threshold = 0.5) and minimal percentage of cells positive for a gene's expression within a cluster set to 25% (min.pct = 0.25) and Wald test was used to evaluate statistical significance. The genes that showed a significant differential expression between a cluster vs. rest of the cells (adj. *P*-value <= 0.05) were deemed to have tissue/cell type-specific expression patterns. Genes which were found to have a cell-type specific expression in blood or ectodermal clusters were removed from further analysis for comparison to congenital heart gene candidates. Heatmaps created using the pheatmap function in R was used to visualize the tissue-specific expression of various genes.

### Histology and Immunohistochemistry

Whole E12.5 and E13.5 embryos were harvested, fixed in 4% paraformaldehyde (Electron Microscopy Sciences) overnight, washed briefly with 1X PBS, and processed using the Leica ASP2065 tissue processor and standard protocol. Tissues were embedded in paraffin and sections collected at a thickness of 6 μm. Staining was performed using Hematoxylin and Eosin (H&E) (Sigma Aldrich) according to the manufacturer's protocol. E13.5 embryos (*n* = 5) were used to examine β-Catenin expression by immunohistochemistry using anti-β-catenin antibody (1:200, Abcam, #ab16051) and anti-rabbit SignalStain Boost IHC Detection reagent (Cell Signaling Technology, 8114). Stained tissue was visualized using a Signal Stain DAB Substrate kit (Cell Signaling Technology, 8059). Sections were washed using TBS containing 0.1% Tween-20.

### Bioinformatics and Statistical Analysis for Human CHD Association Studies

Exome sequencing data generated by the Pediatric Cardiac Genomics Consortium (PCGC) was previously analyzed and published by Jin et al. (21). A patient-specific table of genes with variants was created from the Supplementary data published by Jin et al. and is referenced in this publication. Variants, as reported in Jin et al. are already filtered based on population frequency. We removed synonymous variants from this list. From the remaining dataset, patients diagnosed with tetralogy of Fallot without additional syndromic features (*n* = 419) were examined for *de novo* variants. A list of genes with at least one *de novo* non-synonymous variant amongst the TOF patients was examined for a potential mouse homolog using the BioMart tool in Ensembl and the gene list was named tetralogy of Fallot, *de novo* variant gene with a mouse homolog (TOF-DN-MH) (49). Of the 327 genes with *de novo* variants in the TOF cohort, 270 of them had a mouse homolog that was identified to be expressed in OFT cell types (as determined in our single cell analysis).

We next wanted to determine if the list of 270 genes with *de novo* variants expressed in OFT cell types was enriched for genes that are differentially expressed in the *Notch1*^+/−^*; Nos3*^−/−^ mouse model. Of the 1,352 DEGs from the RNA-Seq analysis of the *Notch1*^+/−^*; Nos3*^−/−^ mouse model, 1,087 of them have human homolog. This list was named differentially expressed genes in *Notch1*^+/−^*; Nos3*^−/−^ OFTs with a human homolog (DEG-HH). The DEH-HH genes were then compared back to the 270 TOF-DN-MH to determine how many genes were in common. A total of 29 genes were found to be in common, i.e., having a *de novo* variant in TOF patients and being differentially expressed in the *Notch1*^+/−^*; Nos3*^−/−^ mouse model.

To determine if these 29 genes represented a significant over-representation (enrichment) of *de novo* variants in genes shown to be differentially expressed in the *Notch1*^+/−^*; Nos3*^−/−^ mouse model, we utilized a test based on the hypergeometric distribution (equivalent to the one-tailed Fisher's exact test) as follows: the number of DEGs with a *de novo* variant represents the overlap (29; q); the total number of human genes with a *de novo* variant and a mouse homolog as group 1 (TOF-DN-MH = 270; m); the total number of known human genes with a mouse homolog represents the population size (16,536 – 270 = 16,266; *n*); the total genes that are differentially regulated in the mouse OFT data which also have a human homolog as group 2 (DEG-HH = 1,054; *k*). The cumulative probability (h) of getting equal or more genes in the overlap was calculated using the phyper function in R as follows:


$$\begin{array}{l} \text{h}=\text{phyper}(\text{q, m, n, k, lower.tail}=\text{F, log.p}=\text{F}) \end{array}$$

We also examined if there is an increased burden of *de novo* missense and loss of function variants amongst these 30 genes using denovolyzeR (50). DenovolyzeR uses a theoretical rate of *de novo* variants estimated from the evolutionary changes between primate and human genomes to determine increased burden of *de novo* variants in patient cohorts. This allows us to examine if there is a significantly higher number of *de novo* loss of function and missense variants in genes in TOF patients compared to a theoretical estimate. We used overlapping genes as the genes to focus on for this analysis.

We also extracted variant information of patients with semilunar valve disease (*n* = 245) and patients diagnosed with hypoplastic left heart syndrome (*n* = 371) using the same parameters described above and named these lists SLV-DN-MH and HLHS-DN-MH, respectively. A total of 19 genes were seen in the SLV-DN-MH intersected with DEG-HH, while a total of 17 genes were seen in the HLHS-DN-MH intersected with DEG-HH. These overlaps were then evaluated using the hypergeometric distribution as described above.

Comparison of the DEG-HH genes with a second cohort of TOF-patients was done using data set from Page et al. (24). Page et al. reported non-synonymous variants filtered using cut-offs in population frequency and CADD scores with damaging effects. However, *de novo* variants and inherited variants were not separated in this dataset. Therefore, this analysis was different from the analysis of *de novo* variants in PCGC. To analyze the 829 TOF patient variants reported in Page et al., we used all the genes that had at least one variant in this dataset and a strong mouse homolog (*n* = 7,907) and named this gene list TOF variant genes (TOF-VAR-PAGE-MH). In order to be consistent between data sets, in parallel, we created a similar gene list with at least one variant (*de novo* and inherited) as reported in Jin et al. and named this list TOF-VAR-JIN-MH (*n* = 1,587). We compared these two patient lists to the DEG-HH list in two steps. In step 1, we examined the individual overlap between the DEG-HH list and TOF-VAR-PAGE-MH gene list and the TOF-VAR-JIN-MH gene list. Then in step 2, we identified common genes that appear in the overlap between these individual comparisons from step 1, focusing on those expressed in OFT cell types. Here, a hypergeometric distribution-based test was applied to calculate the probability of finding common genes amongst Step 1-overlapping genes. The population size was set to the number of DEG-HH genes.

To test whether high expressing genes are predictive of damaging genetic variation in patients with TOF, we selected the top 1,352 genes that showed the highest expression in the OFT RNA seq data in the wildtype embryos and the gene list was named highest expressing mouse outflow tract genes (HEM).This list was of equal number to the DEG gene list in the mouse as to minimize the number of variables between comparisons. The expression, in this case, was determined by calculating the average fragments per kilobase per million reads (FPKM) calculated for each gene in the wildtype dataset. We then overlapped the 270 genes with *de novo* variants identified in the TOF-DN-MH gene list with the HEM gene list and the overlap was then evaluated using a hypergeometric distribution as described above.

## Results

### Characterization of *Notch1^+/−^; Nos3^−/−^* Embryos Reveal Normal Endocardial Cushions

**Figure 1:**
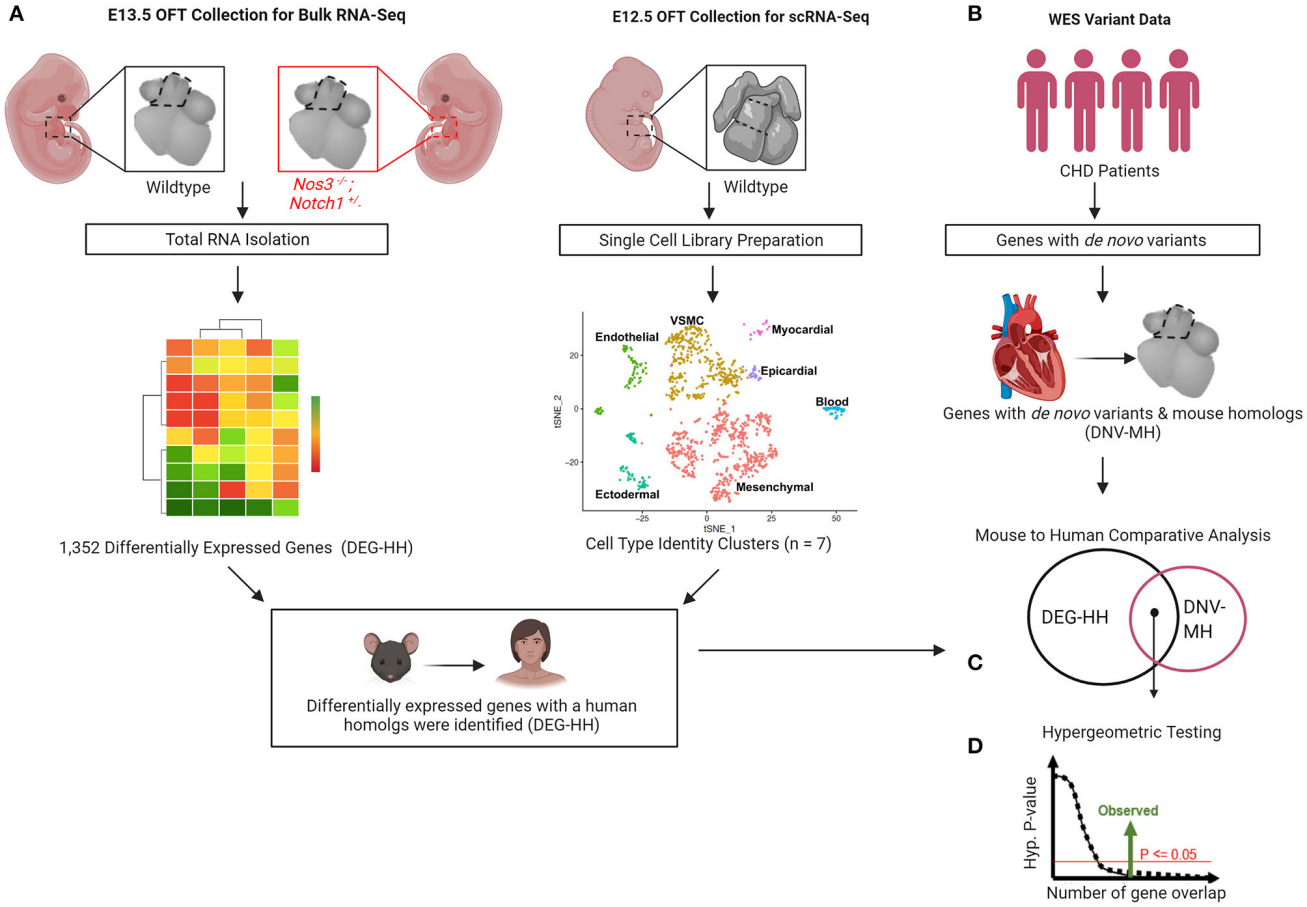
Gene discovery pipeline for human congenital heart disease (CHD) utilizing transcriptomic profiling from wildtype and mutant mouse embryos. **(A)** Differential gene expression by bulk RNA-Sequencing of E13.5 *Notch1*^+/−^*; Nos3*^−/−^ mutant and wildtype outflow tracts (OFT) was performed. **(B)** scRNA-Sequencing transcriptomes derived from wildtype OFT was used to categorize cell identity clusters. **(C)** Differentially expressed genes (DEG) were categorized according to cell identity clusters and human homologs were identified. **(D)** *De novo* gene variants were extracted from WES databases of CHD patients and those with mouse homologs identified. **(E)** Mouse to human comparative analysis with hypergeometric testing was performed to determine statistical significance.

We previously reported a spectrum of cardiac OFT malformations including highly penetrant thickened, malformed semilunar valves and the partially penetrant phenotypes consisting of ventricular septal defects and an overriding aorta in *Notch1*^+/−^*; Nos3*^−/−^ embryos (37, 38). Using the *Notch1*^+/−^*; Nos3*^−/−^ mouse as a genetic tool, we wanted to determine if genes differentially expressed in cells required for the development of the cardiac outflow tract are predictive of damaging *de novo* variants in patients with OFT malformations (Figure 1).

**Figure 2:**
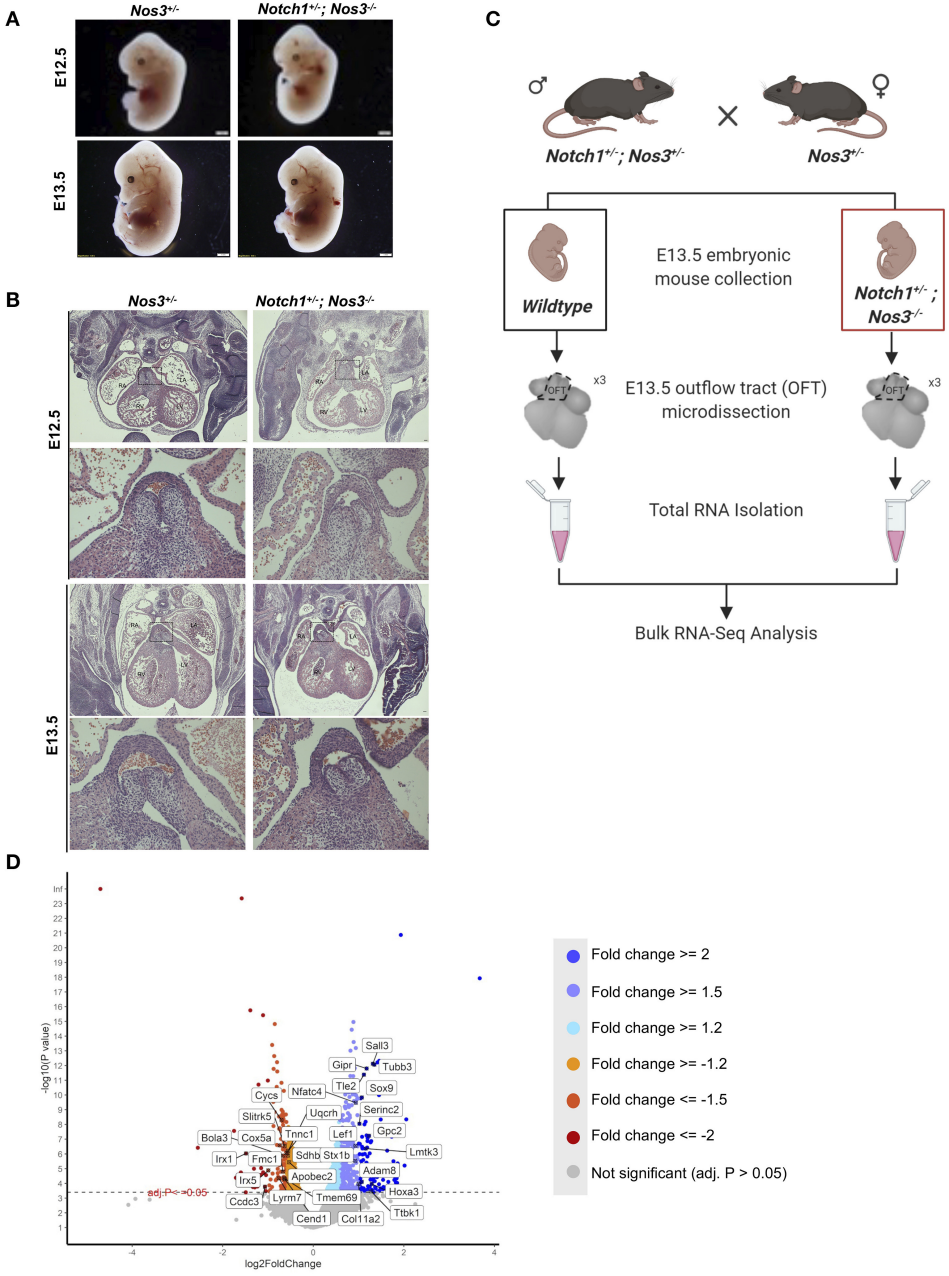
Histologic and RNA-Seq analysis of *Notch1*^+/−^*; Nos3*^−/−^ embryos. **(A)** *Notch1*^+/−^*; Nos3*^−/−^ compound mutant embryos do not show any size differences at E12.5 or E13.5 (*n* = 3). **(B)** Histological section (stained with hematoxylin and eosin) of E12.5 and E13.5 *Notch1*^+/−^*; Nos3*^−/−^ embryonic hearts demonstrates no gross morphological differences. Outflow tract (boxed area in low magnification image) is shown in higher magnification (below) and no differences are noted when compared to *Nos3*^+/−^ littermate controls (*n* = 3). **(C)** Schematic diagram outlining process of OFT collection and bulk RNA-Sequencing from E13.5 *Notch1*^+/−^*; Nos3*^−/−^ and wildtype embryos. **(D)** Volcano plot of 1,352 differentially expressed genes sorted according to fold change and significance (adjusted *p*-value < = 0.05). Significantly downregulated genes with a fold change < −2, < −1.5, < −1.2 are labeled in orange, coral, and red, respectively. Significantly upregulated genes with a fold change > 2, > 1.5, > 1.2 are labeled in turquoise, light purple, and blue, respectively. Highlighted genes are top 15 upregulated and downregulated genes that also have a Notch1 ChIP peak according the previously published Notch1/Rbpjk in mouse ChIP-Sequencing dataset (51). Figure 2C was generated on www.Biorender.com.

Semilunar valve defects are the most predominant phenotype observed in E15.5 and E18.5 compound mutant embryos. First, we compared E12.5 and E13.5 *Notch1*^+/−^*; Nos3*^−/−^ compound mutant embryos to littermate controls (*Nos3*^+/−^) to determine the timing of disease onset. By gross examination, *Notch1*^+/−^*; Nos3*^−/−^ compound mutant embryos at E12.5–E13.5 were grossly normal compared to littermate controls (Figure 2A). By histologic section, the developing outflow tract cushions in compound mutant embryos are indistinguishable from controls at E12.5 and E13.5 (Figure 2B). These results suggest that the onset of semilunar valve disease occurs later in development while the ventricular septal defect with overriding aorta phenotypes are likely the result of earlier developmental abnormalities.

### Transcriptomic Profiling of the *Notch1^+/−^; Nos3^−/−^* OFT Reveals Dysregulation of Multiple Molecular Pathways

**Figure 3:**
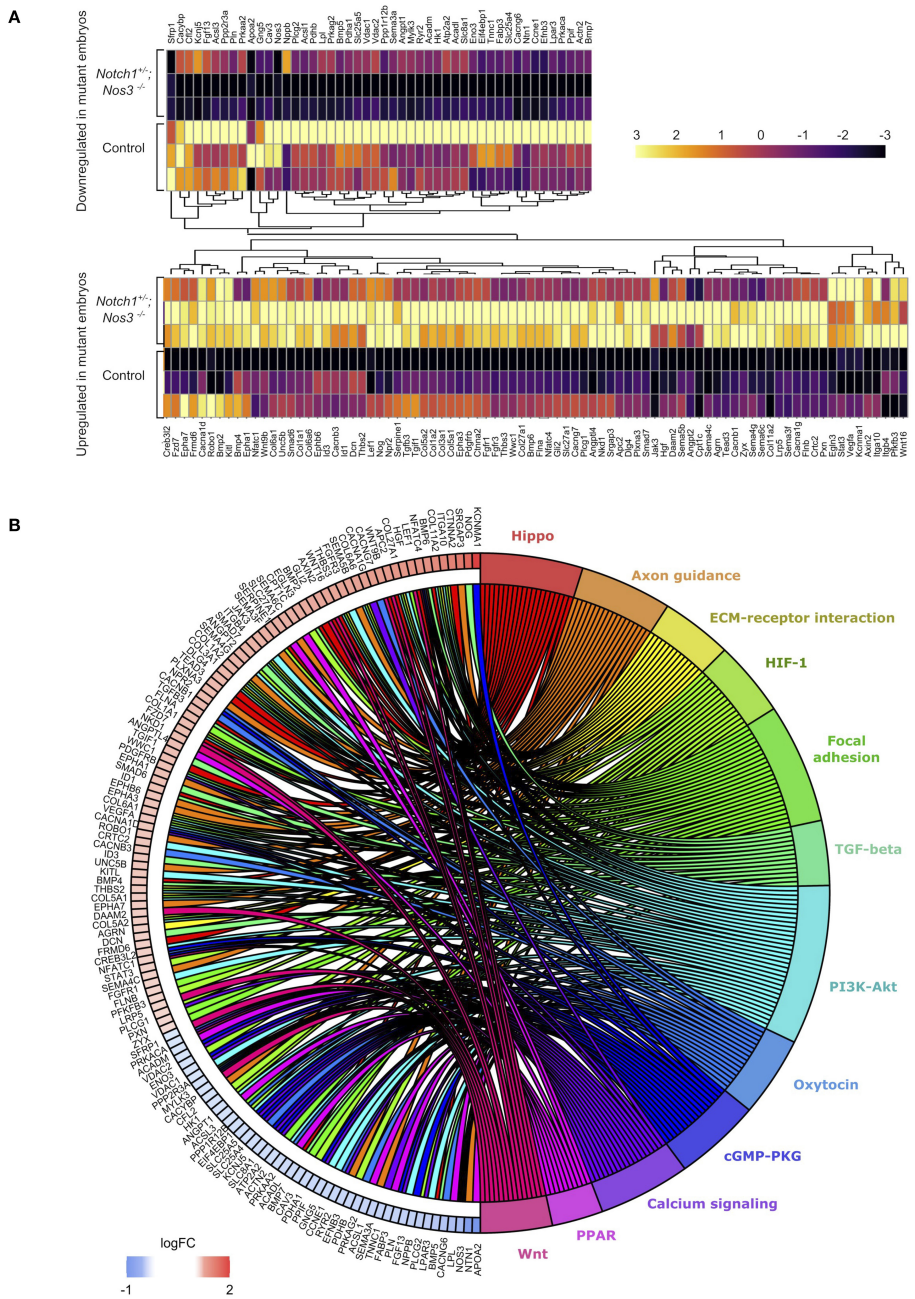
Differential gene expression analysis (DESeq2) shows significant gene expression changes between *Notch1*^+/−^*; Nos3*^−/−^ compound mutants and wildtype (WT) littermate controls. **(A)** Split hierarchical heatmap cluster show differential gene expression among biological replicates obtained from transcriptomic profiling of *Notch1*^+/−^*; Nos3*^−/−^ compound mutants and wildtype littermate controls for a subset of genes (*n* = 3 for each condition). **(B)** Chord plot shows 132 differentially expressed genes identified in bulk RNA-Seq analysis and predicted signaling pathway associations.

We hypothesized that molecular alterations in the OFT of E13.5 *Notch1*^+/−^*; Nos3*^−/−^ compound mutant embryos contribute to the resultant outflow tract malformations found at later timepoints. Accordingly, *Notch1*^+/−^*; Nos3*^+/−^ males were bred to *Nos3*^+/−^ females to obtain E13.5 *Notch1*^+/−^*; Nos3*^−/−^ compound mutants and wildtype (*Notch1*^+/+^*; Nos3*^+/+^) littermate controls. The embryonic OFT was micro-dissected from E13.5 mutant and wildtype embryos, and RNA was isolated for bulk RNA-Seq (Figure 2C). We found 1,352 differentially expressed genes (DEG) which are depicted in the volcano plot (Figure 2D; Supplementary Table 1). Network analysis and visualization using hierarchal heatmap and chord diagrams suggested that *Hif1α*, *Tgf-β*, *Hippo, Wnt* signaling amongst several others were affected in *Notch1*^+/−^*; Nos3*^−/−^ as multiple dysregulated genes were predicted to participate in these pathways (Figures 3A,B) (52, 53). Quantitative RT-PCR of 5 highly DEG, including *Nos3, Netrin-1, Bmp5*, and *Wnt2*, was performed to validate transcriptomic profiling results (data not shown). Among these pathways, we further examined the Wnt signaling pathway as it is a known contributor to endocardial cushion development and myxomatous heart valve disease (54). The consequence of transcriptomic changes in Wnt signaling pathway members can be assessed by inspecting β-catenin protein levels. We found increased staining of β-catenin in the endocardium and endocardial cushions of E13.5 *Notch1*^+/−^*; Nos3*^−/−^ embryos as compared to wildtype controls (Supplementary Figure 1A). Inspection of other disrupted signaling pathways within the *Notch1*^+/−^*; Nos3*^+/−^ OFT is ongoing and beyond the scope of this work. Overall, these results suggest that global disruption of Notch and nitric oxide signaling has potential downstream effects on molecular pathways required for OFT development including *Hif1α*, *Tgf-β*, *Hippo*, and *Wnt* signaling.

### Classification of Bulk Transcriptomic Data Into Seven Cell Identity Clusters by Single-Cell RNA Sequencing (scRNA-Seq)

**Figure 4:**
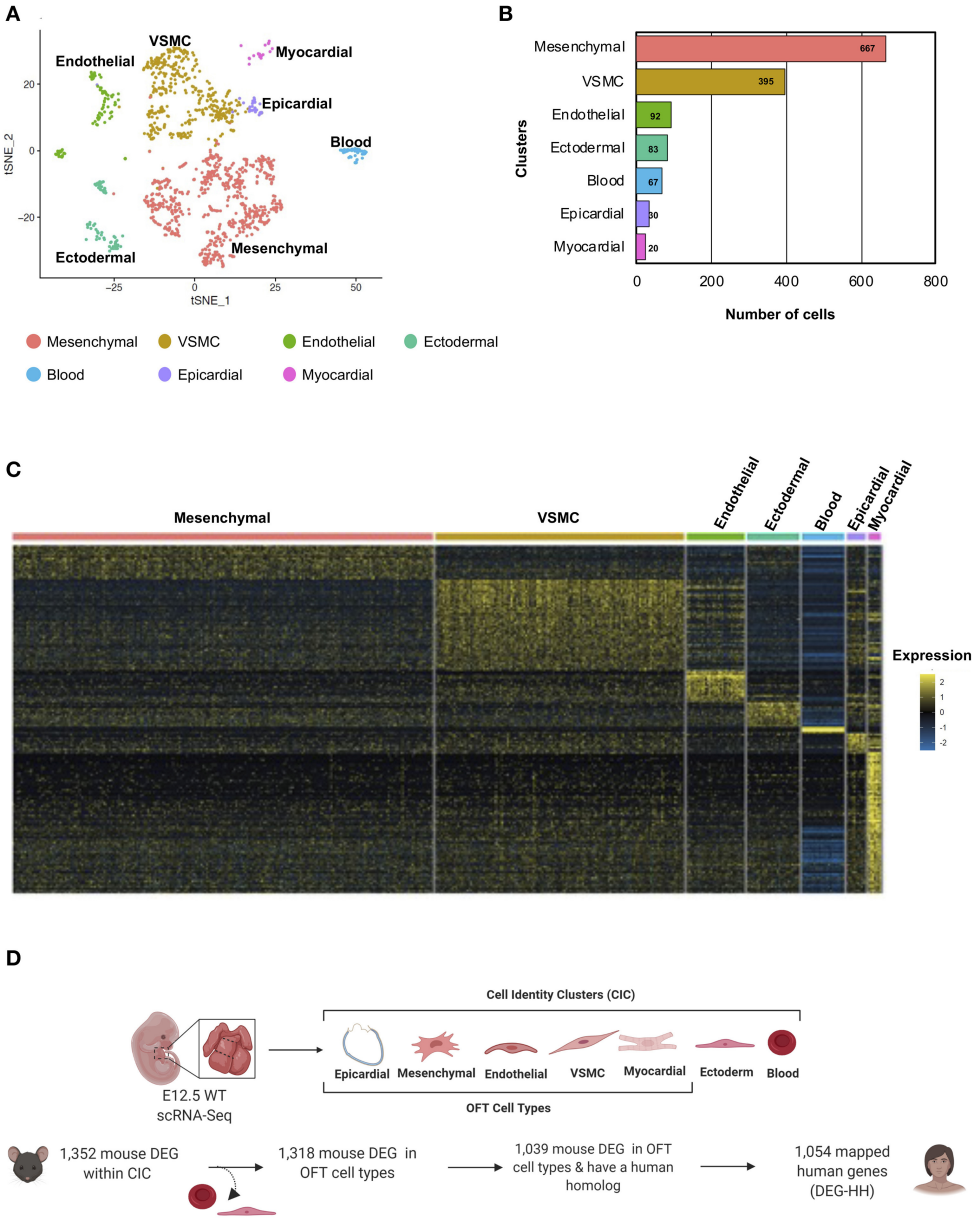
Cellular classification of gene transcripts identified in bulk RNA-Seq utilizing scRNA-Seq analysis of the embryonic cardiac outflow tract. **(A)** tSNE plot of cells captured via scRNA-Seq is shown and classified according to cell identity markers. The identified subclusters are mesenchymal, vascular smooth muscle cells (VSMC), endothelial, ectodermal, blood, epicardial, and myocardial cells. **(B)** Raw count of cells within each cluster indicated that 1,354 cells were captured from the cardiac outflow tract. **(C)** Wildtype gene transcripts identified via bulk RNA-Seq were classified according to scRNA-Seq cell cluster profile; heatmap of overlapping genes is shown. **(D)** Schematic of how the DEG-HH gene list was generated. Differentially expressed genes that were also found to be expressed in the scRNA-Seq dataset were selected. These were filtered based on cell-type specificity and whether they possess a human homolog to generate a candidate list of 1,054 human genes. Figure 4D was generated on www.Biorender.com.

The cardiac OFT is composed of multiple cell types, and in order to determine molecular and cellular pathways which were disrupted in the E13.5 *Notch1*^+/−^*; Nos3*^−/−^ RNA-Seq datasets, we performed scRNA-Seq of the embryonic OFT. scRNA-Seq data generated from pooled E12.5 cardiac OFT was readily available to us and was used for subsequent analysis. Following pre-processing steps, which revealed 1,354 cells were captured within the sample, tSNE visualization was performed and clustering analysis found nine distinct groups within the wildtype E12.5 OFT (Figure 4A). Clustering annotation was performed by finding the gene signature of each cluster using marker genes that delineate cell identities (55–59). The clusters were reduced and classified as mesenchymal, vascular smooth muscle, endothelial, ectodermal, epicardial, myocardial, and blood cells (Figure 4B; Supplementary Figure 1B). Next, we re-analyzed the 1,352 gene transcripts identified in the bulk RNA-Seq to be DEG according to the scRNA-Seq gene cluster identities in which they are predominantly expressed (Figure 4C). The DEG transcripts (1,039) with a human homolog (1,054) were categorized by cell type, due to mouse transcripts mapping to multiple homologous human genes (Figure 4D). In parallel, we examined the tissue-specific expression of the 1,352 DEG genes. We found 431 of these mouse genes preferentially expressed amongst the seven cell identity clusters. Those that were also differentially expressed in *Notch1*^+/−^*; Nos3*^−/−^ compound mutant embryos are referred henceforth as DEG mouse gene list (Supplementary Tables 1, 2). This analysis demonstrated disruption of molecular pathways in each of these cell types (myocardial, mesenchymal, VSMC, endothelial) that populate the developing OFT.

### Differentially Expressed Genes in *Notch1^+/−^; Nos3^−/−^* Mouse OFT Are Enriched as *de novo* Variants in TOF Patient Cohorts

**Figure 5:**
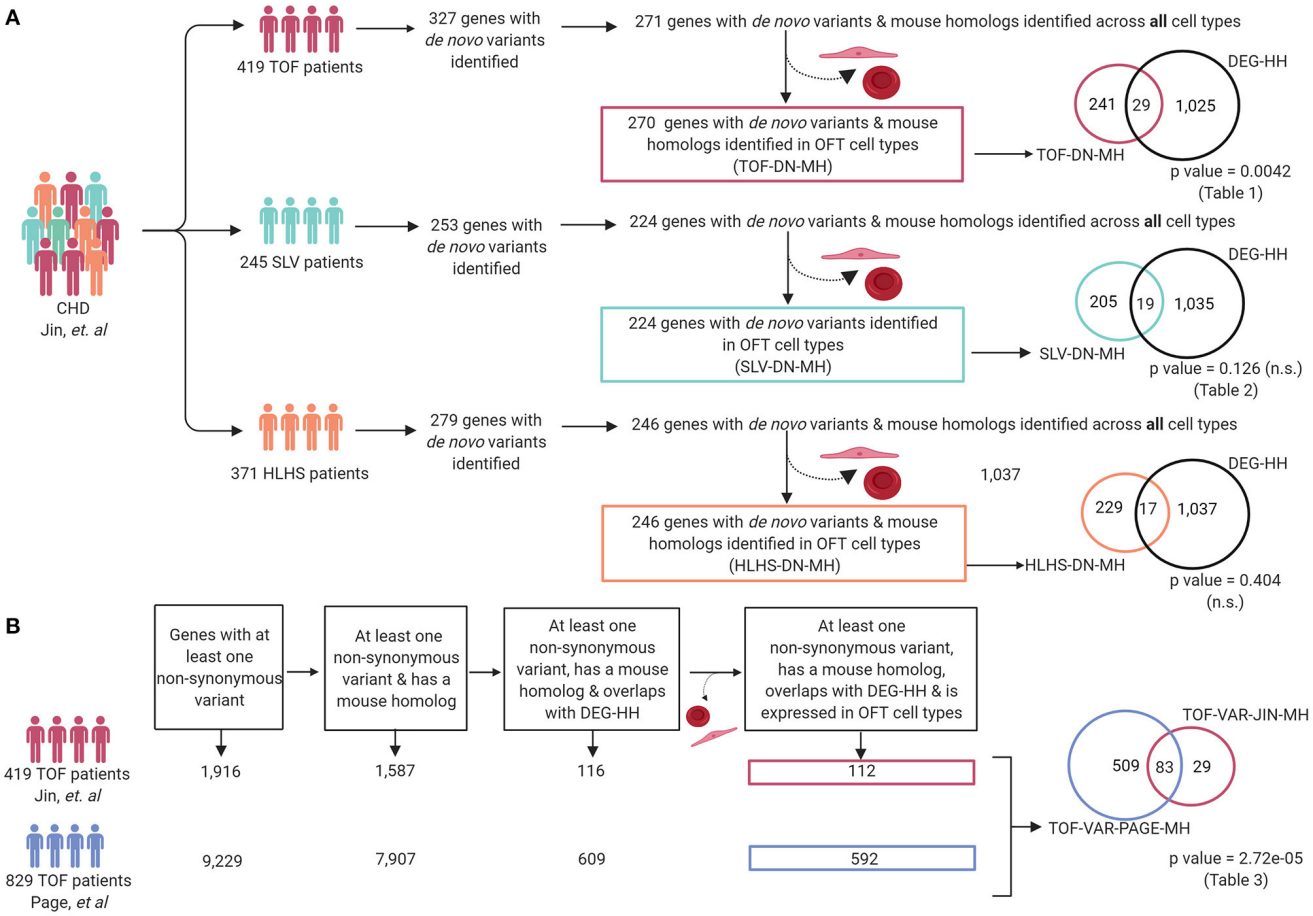
Differentially expressed genes in *Notch1*^+/−^*; Nos3*^−/−^ compound mutants are enriched within reported genetic variants from TOF but not SLV and HLHS patients. **(A)** Patients with a primary TOF and SLV diagnosis and their associated *de novo* variant information were extracted from Jin et al. and compiled for analysis (TOF-DN-MH and SLV-DN-MH, respectively). Overlapping of TOF-DN-MH and SLV-DN-MH with DEG-HH demonstrated a significant enrichment of genes between TOF-DN-MH and DEG-HH but not SLV-DN-MH (*p*-value = 0.0042 and *p*-value = 0.126, respectively). Overlapping of the HLHS-DN-MH cohort with DEG-HH failed to reach statistical significance (*p*-value = 0.404). **(B)** Damaging, non-synonymous variants specific to TOF patients were extracted from Page et al. and Jin et al. and assessed for mouse gene homologs and compiled for analysis (TOF-VAR-PAGE-MH & TOF-VAR-JIN-MH, respectively). After overlapping with differentially expressed genes in the *Notch1*^+/−^*; Nos3*^−/−^ compound mutants and assessing those genes found in OFT cell types, we found 83 shared genes among the 112 (TOF-VAR-JIN-MH) and 592 (TOF-VAR-PAGE-MH) genes in each cohort (*p*-value = 2.72e-05). Figure was generated on www.Biorender.com.

A large number of patients with CHD have undergone genomic sequencing and numerous potential genetic contributors have been reported (60). We asked if differentially expressed genes in a mouse model of cardiac OFT malformations overlapped with identified putative disease-causing variants reported from large cohort studies. We selected 419 patients from the Pediatric Genomics Consortium (PCGC) published in Jin et al. with a primary diagnosis of TOF (Supplementary Table 3A). We examined the rare *de novo* variants (population frequency < = 0.05) found in these 419 TOF patients (23). To compare this data to the mouse model, we retained only those genes with a *de novo* variant and a mouse homolog (TOF-DN-MH), which numbered 271. This was filtered further to 270 by selecting against genes that are predominantly expressed in ectoderm and blood as developing ectoderm and blood cells are not known to contribute to development of the OFT or TOF (Supplementary Table 1). From the mouse model, we selected 1,352 significantly impacted (adjusted *P*-values < = 0.05) transcripts from the *Notch1*^+/−^*; Nos3*^−/−^ OFTs and identified its human homolog (DEG-HH, *n* = 1,054) expressed in OFT cell types (Figure 4D). By assessing genes expressed in OFT cell types (myocardial, epicardial, mesenchymal, endothelial, and VSMC) for genetic homologs, we found an enrichment of *de novo* gene variants in the TOF-DN-MH population and the DEG-HH gene lists as according to statistical analysis (hypergeometric *p*-value = 0.0042). Ultimately, we found 29 genes with *de novo* variants that are likely contributors of TOF considering they are dysregulated in a mouse model of OFT-disease, present as damaging, *de novo* variant carrying genes in this CHD genomic dataset and predominantly expressed in OFT cell types (Figure 5A; Table 1).

**Table 1:** Overlap between DEG-HH and TOF-DN-MH gene lists.

**Human gene name**	**Murine Ensembl Gene ID**	**Lethality (Embryonic/Neonatal/Perinatal)**	**Murine cardiovascular development phenotype**	**References**
*ACSL1*	ENSMUSG00000018796	(–)	(+)	(61, 62)
*ATP2A2*	ENSMUSG00000029467	(+)	(+)	(63)
*CLK1*	ENSMUSG00000026034	(–)	(–)	(64)
*DCLK1*	ENSMUSG00000027797	(–)	(–)	(65)
*DDR2*	ENSMUSG00000026674	(–)	(+)	(66)
*EEPD1*	ENSMUSG00000036611	(–)	(–)	MGI:4415486
*FAM110B*	ENSMUSG00000049119	(–)	(–)	(64)
*FAT4*	ENSMUSG00000046743	(+)	(+)	(67)
*FBN2*	ENSMUSG00000024598	(+)	(–)	(68)
*GAN*	ENSMUSG00000052557	(–)	(–)	(69)
*HDAC7*	ENSMUSG00000022475	(+)	(+)	(70, 71)
*JAG2*	ENSMUSG00000002799	(+)	(+)	(72)
*KIF5A*	ENSMUSG00000074657	(+)	(–)	(73)
*MAPK8IP3*	ENSMUSG00000024163	(+)	(–)	(74)
*MAPRE2*	ENSMUSG00000024277	(–)	(–)	MGI:106271
*MED13L*	ENSMUSG00000018076	(+)	(+)	(75)
*MLF1*	ENSMUSG00000048416	(–)	(–)	(76)
*MYH6*	ENSMUSG00000040752	(+)	(+)	(77)
*MYOM2*	ENSMUSG00000031461	(–)	(–)	MGI:6114782
*NOTCH1*	ENSMUSG00000026923	(+)	(+)	(39)
*PKP2*	ENSMUSG00000041957	(+)	(+)	(78)
*PRICKLE3*	ENSMUSG00000031145	(–)	(–)	MGI:4455994
*SEMA3A*	ENSMUSG00000028883	(+)	(+)	(79)
*SMAD6*	ENSMUSG00000036867	(+)	(+)	(80, 81)
*SNAI1*	ENSMUSG00000042821	(+)	(+)	(82)
*THBS2*	ENSMUSG00000023885	(–)	(+)	(83)
*TMTC2*	ENSMUSG00000036019	(–)	(–)	MGI:5319875
*TRIM63*	ENSMUSG00000028834	(+)	(+)	(84)
*VCAN*	ENSMUSG00000021614	(+)	(+)	(85)

*Genes that were identified to be differentially expressed in E13.5 Notch1^+/−^; Nos3^−/−^ cardiac OFT and possess de novo variants in patients with TOF are listed. Of the 29 genes identified, murine lethality and cardiovascular development phenotypes are listed. (+) indicates that lethality (embryonic, neonatal, or perinatal) or cardiovascular development phenotypes have been previously reported in murine models. (–) indicates that lethality or cardiovascular phenotypes have not been previously reported or observed*.

Among the identified genes, several have been previously implicated to contribute to outflow tract development in mice. Not surprisingly, members of the Notch signaling pathway, specifically *Notch1* and *Jag2*, were identified to be differentially expressed in mice and identified to possess damaging, *de novo* variants in TOF patients (38, 39, 72, 86). Genes required for endocardial cushion development in mice *Snai1, Vcan*, and *Smad6* were also identified as strong candidates (7, 80, 82). Genes not previously implicated in the development of the cardiac outflow tract yet recognized to contribute to other aspects of heart development were identified including *Myh6, Pkp2, Sema3a, Fat4, Hdac7, Ddr2, Med13l, and Acsl1* (66, 67, 70, 71, 75, 77–79, 87–89). Literature review of the remaining gene candidates found no CHD phenotypes having been described in existing mouse models.

Considering *Notch1*^+/−^*; Nos3*^−/−^ mice display a wide range of SLV malformations, we repeated the previously outlined analysis on 245 patients from PCGC with CHD affecting the semilunar valves, aorta and aortic arch arteries (encompassed the following phenotypes: aortic and pulmonary valve stenosis, patent ductus arteriosus, aortic arch artery malformations, bicuspid, and unicuspid aortic valve) (Figure 5A; Supplementary Table 3B). In repeating the analysis outlined previously for TOF patients, we identified 224 genes with *de novo* variants present in SLV disease populations in OFT cell types, 19 of which overlapped with the DEG-HH list. This overlap did not reach statistical significance (hypergeometric *p*-value = 0.126) (Figure 5A; Table 2).

**Table 2:** Overlap between DEG-HH and SLV-DN-MH gene lists.

**Human gene name**	**Mouse gene name & Ensembl Gene ID**	**Lethality (Embryonic/Neonatal/Perinatal)**	**Murine cardiovascular development phenotype**	**References**
*CCDC82*	ENSMUSG00000079084	(–)	(–)	
*CEP250*	ENSMUSG00000038241	(–)	(+)	(90)
*GIGYF1*	ENSMUSG00000029714	(–)	(–)	
*GPR162*	ENSMUSG00000038390	(–)	(–)	MGI:3797526
*IGDCC4*	ENSMUSG00000032816	(–)	(–)	
*KANK1*	ENSMUSG00000032702	(–)	(–)	MGI:6257642
*KCNJ5*	ENSMUSG00000032034	(–)	(+)	(91)
*LRP1*	ENSMUSG00000040249	(+)	(+)	(92)
*MYH11*	ENSMUSG00000018830	(+)	(+)	(93)
*MYOF*	ENSMUSG00000048612	(–)	(–)	(94)
*NLRC3*	ENSMUSG00000049871	(–)	(–)	(95)
*NOTCH2*	ENSMUSG00000027878	(+)	(+)	(96)
*NPHP3*	ENSMUSG00000032558	(+)	(+)	(97)
*PSME1*	ENSMUSG00000022216	(–)	(–)	N.K.
*PTPRU*	ENSMUSG00000028909	(–)	(–)	MGI:5608701
*TGM2*	ENSMUSG00000037820	(–)	(+)	(98, 99)
*UQCRC*	ENSMUSG00000025651	(+)	(–)	(100)
*RYR2*	ENSMUSG00000021313	(+)	(+)	(101–103)
*UNC5B*	ENSMUSG00000020099	(+)	(+)	(104)

*Genes that were identified to be differentially expressed in E13.5 Notch1^+/−^; Nos3^−/−^ cardiac OFT and possess de novo variants in patients with SLV are listed. Of the 19 genes identified, murine lethality and cardiovascular development phenotypes are listed. (+) indicates that lethality (embryonic, neonatal, or perinatal) or cardiovascular development phenotypes have been previously reported in murine models. (–) indicates that lethality or cardiovascular phenotypes have not been previously reported or observed*.

To determine the utility of our mouse to human comparative analysis pipeline, we performed a secondary analysis using a distinct cohort consisting of 829 non-syndromic TOF patients described by Page et al. as described previously with minor modifications (Figure 5B; Supplementary Table 3D) (24). As the inheritance of these variants was not available, we generated a list of genes that were reported to possess at least one non-synonymous variant and also had a mouse homolog (TOF-VAR-PAGE-MH). Then, we overlapped the TOF-VAR-PAGE-MH gene list with the DEG-HH list and to generate a filtered list of 592 candidates that were identified to have non-synonymous variants in patients with TOF, possess a mouse homolog, be differentially expressed in *Notch1*^+/−^*; Nos3*^−/−^ mouse OFT and expressed in OFT cell types. In parallel, we generated a candidate gene list using TOF patient data derived from Jin et al. that included genes all non-synonymous variants (*de novo* and inherited) that also possessed a mouse homolog (TOF-VAR-JIN-MH). We overlapped the TOF-VAR-JIN-MH gene list with the DEG-HH and assessed for expression in OFT cell types as described previously to generate a filtered list of 112 candidates. We then examined whether there was a significant overlap of genes with all non-synonymous variants, regardless of inheritance, between both TOF cohorts (TOF-VAR-PAGE-MH vs. TOF-VAR-JIN-MH) that were also identified to be differentially expressed in *Notch1*^+/−^*; Nos3*^−/−^ mouse OFT. In doing so, we identified 83 shared genes which reached statistical significance (2.72 × 10^−5^) (Table 3; Figure 5B). Of these identified genes, 13 were associated with a cardiovascular phenotype as determined by the Online Mendelian Inheritance in Man compendium which includes *ABCC9, CENPF, HADHA, HSPA9, MED13L, MIPEP, MYH6, MYPN, NEXN, NOS3, NOTCH1, SMAD6*, and *TNNT2*. Thirty-six of these genes are not yet linked to human disease; 18 of which were previously linked to cardiovascular development in various animal modeling systems.

**Table 3:** Overlapping genes between TOF-VAR-PAGE-MH and TOF-VAR-JIN-MH lists.

**Human gene name**	**OMIM**	**Human gene name**	**OMIM**	**Human gene name**	**OMIM**	**Human gene name**	**OMIM**
ABCC9*	601439	*FBN2*	612570	*NDUFA13*	609435	TNNT2*	191045
*ADAMTS20*	611681	*FLNB*		*NDUFS3*	603846	*TRIM46*	600986
*ADAMTS8*	605175	*FMNL3*	616288	NEXN*	613121	*UBA7*	
*ADPRHL1*	610620	*FSD2*		NOS3*	163729	*UQCC1*	611797
*AGRN*	103320	*GAN*	605379	NOTCH1*	190198	*UQCRC2*	191329
*ANGPT2*	601922	*GLI2*	165230	*NOTCH2*	618026	*VCAN*	118661
*ANGPTL4*	605910	HADHA*	600890	*PAM*	170270	*WSB1*	610091
*ANKRD24*		*HDAC7*	606542	*PCCA*	232000	*ZSWIM8*	
*AS3MT*	611806	HSPA9*	600548	*PDLIM3*	605889		
*ATP2A2*	108740	*ISLR*	602059	*PLEKHA6*	607771		
CENPF*	600236	*ITGA10*	604042	*PLEKHG2*	611893		
*CEP170B*		*JAG2*	602570	*POLG2*	604983		
*CLK1*	601951	*KIF26A*	613231	*PRICKLE3*	300111		
*CMYA5*	612193	*LARP7*	612026	*PRKAR2A*	176910		
*COL1A1*	120150	MED13L*	608771	PTGIS*	601699		
*COL6A1*	120220	MIPEP*	602241	*RNASET2*	612944		
*COL6A6*	616613	*MLF1*	601402	*SEMA3A*	603961		
*COL9A1*	120210	*MRPL19*	611832	*SFXN3*	615571		
*CRAT*	600184	*MTHFR*	236250	*SH3PXD2A*			
*DCLK1*	604742	MYH11*	160745	SMAD6*	602931		
*DDR2*		MYH6*	160710	*SUCLG2*	603922		
*EEPD1*	617192	*MYH7B*	609928	*SYNPO*	608155		
*FAM110B*	611394	MYO3B	610040	*THBS2*	188061		
*FAM189B*		*MYOM2*	603509	*TLE3*	600190		
*FAT4*	612411	*MYPN*	608517	*TMTC2*	615856		

Genes that were identified to be differentially expressed in E13.5 Notch1^+/−^; Nos3^−/−^ cardiac OFT and possess overlapping non-synonymous variants in patients with TOF across two distinct cohorts are listed. Genes previously associated with cardiovascular disease according the Online Mendelian Inheritance in Man (OMIM) database are indicated using (*).

To test the validity of our gene prioritization pipeline, we performed two separate bioinformatic analyses. First, we repeated mouse to human comparative analysis in a non-OFT malformation population (Figure 5A; Supplementary Table 3C). Patients with hypoplastic left heart syndrome (HLHS) are characterized by a hypoplastic left ventricle, aorta, and mitral valve. HLHS is recognized to be an oligogenic and genetically heterogeneous type of CHD therefore, we hypothesized there would be minimal to no overlap between genes with *de novo* or inherited variants and the DEG-HH gene lists generated by our OFT-disease model than expected by chance (55). As expected, there was no more overlap of genes (*n* = 17) between these gene lists than by chance suggested by our statistical test (hyper *p*-value = 0.39).

**Figure 6:**
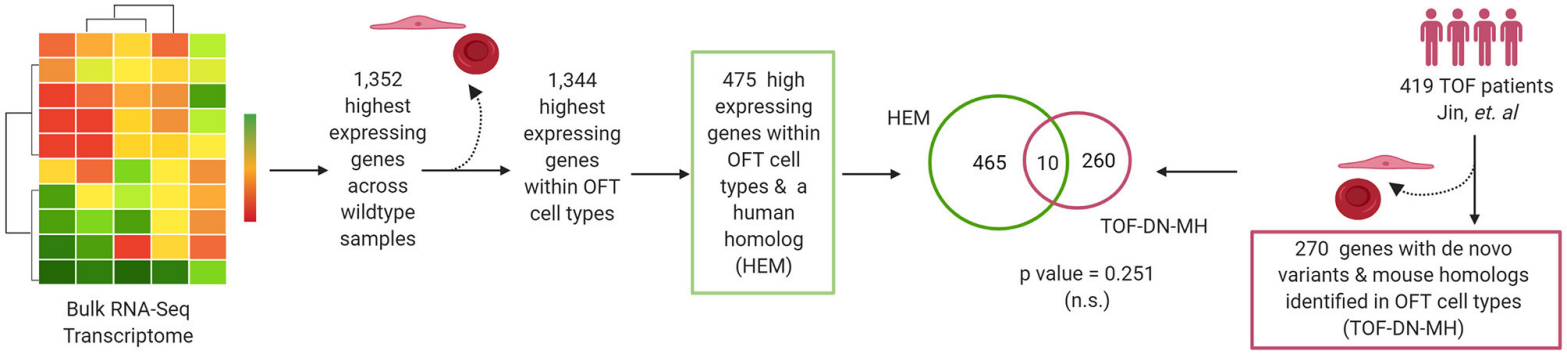
Highest expressed genes in cardiac outflow tract identified by RNA-Seq are not predictors of *de novo* variants in tetralogy of Fallot (TOF) cohorts. The highest expressing genes (*n* = 1,352) across all three wildtype mouse outflow tract (OFT) samples were selected. Of these (*n* = 475) were expressed with OFT cell types and also possessed a human homolog. We assessed the overlapped the TOF-DN-MH gene list with the HEM gene list in OFT cell types and determined no enrichment of genes between these datasets (*p*-value = 0.251). Figure was generated on www.Biorender.com.

Previous research identified enrichment of damaging *de novo* mutations in patients with multiple CHD diagnoses in genes considered to be highly expressed in the developing heart as this was used by the PCGC (105). Using the bulk RNA-Seq transcriptome, we selected the top 1,352 genes with the highest average RPKM across all three sequenced wild-type OFT samples, identified their genetic homolog, and determined whether there was the overlap of genes with *de novo* mutations in the TOF-DN-MH variant list and genes highly expressed in mouse (HEM) OFT (Figure 6; Supplementary Table 4). We were unable to identify any more genes with *de novo* mutations identified in this comparative analysis than what was expected by chance (*n* = 10). Removal of genes expressed primarily in ectodermal, and blood cells from the HEM OFT gene lists did not improve this association. Overall, our results demonstrate that mouse to human comparative analysis can identify with statistical significance genes with *de novo* variation in distinct CHD populations.

## Discussion

By using the differentially expressed transcriptome from a mouse model of CHD, we demonstrate a methodology that may allow for improved classification of potential genetic contributors that are generated from exome sequencing of patients with CHD. *Notch1*^+/−^*; Nos3*^−/−^ mice are a highly penetrant model of cardiac OFT malformations. Differential gene expression and network analysis of E13.5 *Notch1*^+/−^*; Nos3*^−/−^ OFT showed that multiple genes and pathways were disrupted downstream of *Notch1* and *Nos3* including the *Hif1α*, *Tgf-β*, *Hippo*, and *Wnt* signaling pathways. We reclassified wildtype gene transcripts identified in the bulk RNA-Seq according to cellular identity clusters generated from wildtype scRNA-Seq data. In doing so, we generated a list of candidate genes expressed in known cellular contributors of the developing OFT. We found that genes that were differentially expressed in E13.5 *Notch1*^+/−^*; Nos3*^−/−^ OFT (DEG-HH) are present as damaging, *de novo* variants in patients diagnosed with TOF. There was specificity of our pipeline as it failed to detect a significant enrichment of genes with *de novo* variants in a HLHS population. We were also unable to detect an enrichment of genes with *de novo* mutations in the TOF-DN-MH variant list when compared to gene lists generated from the highest expressing genes in the mouse OFT. In summary, these findings highlight the value of utilizing transcriptomic profiling datasets from highly penetrant mouse models of disease when attempting to determine the clinical significance of genetic variants identified by large scale sequencing efforts of patients with CHD (Figure 1).

Our current understanding of definitive genetic contributors of OFT-derived malformations is limited to familial inheritance studies, sequencing of large populations of affected individuals, and mouse modeling approaches. TOF can occur in isolation (non-syndromic) as well in combination with non-cardiac anomalies (syndromic). Syndromic TOF accounts for ~20% of cases (e.g., 22q11.2 deletion syndrome) while the genetic contributors of non-syndromic TOF are not entirely elucidated. Single-gene knockout studies in mice have been instrumental in describing in detail the morphological changes that occur in the developing OFT following gene disruption. Oligogenic disruptions are also known to contribute to heart disease as noted by exome sequencing of trios and compound mouse mutant modeling (106). Through this publication, we have been able to demonstrate the benefit of a mouse to human comparative analysis to prioritize genes candidates previously understudied in the development of non-syndromic TOF. Global and conditional deletion mouse strains are available for many of the prioritized gene candidates identified in this publication. However, many of these candidates have not been studied in the context of cardiac OFT development which hinders our understanding of their functional role in the heart. Similarly, while exome sequencing approaches have identified potential damaging contributors of high statistical significance, these studies lack *in vivo* validation using animal models which limits the application of these findings in a clinical setting. We propose that mouse models of disease, such as the *Notch1*^+/−^*; Nos3*^−/−^ compound mutant mouse line, are instrumental tools as they provide much needed *in vivo* evidence of gene candidates identified through large-scale sequencing screens.

We recognize that there are limitations to our work that hinder our ability to make more definitive and potentially more clinically relevant conclusions. First, although *Notch1*^+/−^*; Nos3*^−/−^ animals are recognized to be a highly penetrant model of OFT malformations, the phenotype observed is quite variable. The predominant phenotype observed is SLV stenosis however TOF-like phenotypes are also observed at a lower rate. Therefore, we suspected that transcriptomic profiles generated from E13.5 *Notch1*^+/−^*; Nos3*^−/−^ OFTs would differ between sequenced samples. Accordingly, we did note that one of the three samples sequenced was substantially different from the other two. In lieu of removing available RNA-sequencing data, we decided it was best to proceed with *n* = 3 datasets despite the variability observed as one could argue that disease observed between CHD patients harboring the same genetic mutation is also highly variable. To our knowledge, there is no perfect animal model for OFT malformations, and we argue that the identification of dysregulated genes in murine models of CHD could provide sufficient functional evidence to better assess variants of unknown significance identified by genetic sequencing of affected populations. Furthermore, these type of analyses offer initial observations to stimulate the study novel genetic contributors of OFT development as compared to making use of transcriptomic data derived from unaffected animals with normal hearts. Second, we recognize that we may have selected too late of a time point in the development of the cardiac OFT to investigate potential contributors of TOF. At E13.5 the location of the great vessels has already been established; one of the hallmarks of TOF is a displacement of the aorta. Similarly, E13.5 SLV has already undergone EMT, although valve remodeling and elongation has not yet occurred. It is possible that by performing transcriptomic profiling of E13.5 *Notch1*^+/−^*; Nos3*^−/−^ OFTs we only detected the tail-end expression of critical developmental pathways required for the development of the great vessels and EMT of the semilunar valve, which may affect our downstream analysis. However, we were able to detect several genes in patients with TOF that were also differentially expressed in mouse OFT which suggests our findings are of clinical relevance. Future studies using cross-species analysis should take into consideration the developmental milestones that occur prior to the onset of disease before performing a transcriptomic analysis of mouse models. Another limitation was the use of an scRNA-seq E12.5 OFT library as opposed to an E13.5 timepoint. Currently, there is no publicly available single-cell RNA-seq data set of the E13.5 OFT. Developmentally, the E12.5 and E13.5 OFT are similar considering EMT is still underway at both timepoints and the contributions of the cardiac neural crest and second heart field to OFT septation are complete. Therefore, while not the ideal we do not believe the classification of transcripts is significantly affected by this process nor does it impact downstream analysis. Finally, considering we selected variant information derived from publicly available databases we are limited to the information presented in these reports and unable to validate the bioinformatic conclusions we generated here. However, given the size of the patient populations selected and the filtering criteria used to call damaging genetic variation we are confident in our analysis as a method to identify causative genes involved in OFT malformations. Future work must focus on validating the expression and functionality of these gene products in developing hearts.

With the growing availability of exome and genome sequencing technologies, there is expected to be an increase in the use of this method of genetic testing in CHD populations. However, sequence variants identified in these patients are often classified as variants of uncertain significance (VUS) as opposed to being deemed pathogenic due to limited availability of relevant biological data. Barring very few examples, where the specific variants identified in human OFT malformation have been modeled in mice, there has been limited evaluation of the effect of the identified human sequence variant on the development of the OFT (41, 107). This has not only hampered the classification of novel variants as pathogenic or benign but also limits the establishment of clear links between CHDs and novel candidate genes identified in large patient cohort studies. One way to prioritize and strengthen the genetic link between novel candidate genes and CHDs is to determine if their expression is altered during the development of OFT in an animal model of the disease. As clinical databases become more enriched with patient sequencing information, transcriptomic profiling of disease models has the potential to provide additional data to assist in the classification of sequence variants. Our work demonstrates the utility of using a disease specific model to generate transcriptomic profiling data for the purposes of identifying genes of clinical significance in patients with TOF. Accordingly, we describe a pipeline that may improve the analysis of genetic sequencing data from human patient cohorts and overlay pathological relevance from disease models to purely bioinformatic findings.

## Data Availability Statement

The original contributions presented in the study are included in the article/Supplementary Material. Single cell RNA-Sequencing data has been deposited in GEO under accession number GSE171239. Further inquiries can be directed to the corresponding author.

## Ethics Statement

The animal study was reviewed and approved by Institutional Animal Care and Use Committee at Nationwide Children's Hospital.

## Author Contributions

The experimental data was collected by AM-N and UM. Bioinformatics data analysis was performed by SM. Data analysis was reviewed and evaluated by AM-N, UM, SM, KM, PW, and VG. Original draft of the manuscript was generated by AM-N with contributions from SM and VG. All authors contributed to the designing of the experiments, drawing conclusions from the data, and editing of the manuscript.

## Author Disclaimer

The content is solely the responsibility of the authors and does not necessarily represent the official views of the National Institutes of Health.

## Conflict of Interest

The authors declare that the research was conducted in the absence of any commercial or financial relationships that could be construed as a potential conflict of interest.

## Publisher's Note

All claims expressed in this article are solely those of the authors and do not necessarily represent those of their affiliated organizations, or those of the publisher, the editors and the reviewers. Any product that may be evaluated in this article, or claim that may be made by its manufacturer, is not guaranteed or endorsed by the publisher.
