# Supplementary material for: A Multi-Omics Approach Using a Mouse Model of Cardiac Malformations for Prioritization of Human Congenital Heart Disease Contributing Genes
Source: Front Cardiovasc Med. 2021 Aug 24;8:683074. doi: 10.3389/fcvm.2021.683074 (PMC8421733; doi:10.3389/fcvm.2021.683074)
Supplement: Supplementary file 2 [file Data_Sheet_2.docx]

**SUPPLEMENTAL MATERIALS**

**Supplemental Figure Legends**

**Supplemental Figure 1. Validation of molecular differences in *Notch1^+/-^; Nos3^-/-^* mutant embryos and cell identity analysis of scRNA-seq.** **(A)** Immunohistochemistry shows upregulation of β-catenin in the cardiac outflow tract of E13.5 *Notch1^+/-^; Nos3^-/-^* compound mutants when compared to wildtype littermate controls (n=5). Arrow highlights the endocardial cell layer of the outflow tract cushion. **(B)** Heatmap of scRNA-Seq data cluster and associated gene markers used to delineate cell identities (left).

**Supplemental Table 1.** ***Notch1^+/-^; Nos3^-/-^* DESeq Bulk RNA-Seq Analysis**

Genes (1,352) identified to be differentially expressed in bulk RNA-Seq are listed (Ensemble Gene ID & Mouse Gene Symbol) and ranked according to fold change and adjusted p-values. Human homologs of DEG are listed; these are filtered according to cell-type expression (DEG-HH gene list). Genes with *de novo* variants in TOF and SLV cohorts (Jin, *et. al.*) are listed and are filtered according to DEG-HH resulting in TOF-DN-MH and SLV-DN-MH, respectively.

**Supplemental Table 2.**  **DEG identified via bulk RNA-Seq classified according to scRNA-Seq clusters**

Genes identified to be differentially expressed in bulk RNA-Seq were classified according to seven scRNA-Seq clusters which included mesenchymal, vascular smooth muscle, endothelial, ectodermal, epicardial, myocardial, and blood cells. To differentiate gene expression between cells, average log fold change (Average logFC) was calculated. A minimum percentage of cells positive for a gene’s expression within a cluster (pct) was required to assign a gene to cluster cell identity. Genes that showed a significant differential expression between a cluster vs rest of the cells (adj.P-value <= 0.05) were deemed to have tissue/cell type-specific expression patterns. Duplicate genes across distinct cell types are labelled in red.

**Supplemental Table 3.** (3A) TOF Cohort reproduced from Jin, et. al. (Nat Gen, 2017)

(3B) SLV Cohort reproduced from Jin, et. al. (Nat Gen, 2017) (4C) HLHS Cohort reproduced from Jin, et. al. (Nat Gen, 2017) (3D) TOF cohort reproduced from Page, et. al. (Circulation Research, 2019)

**Supplemental Table 4.**  **Highest expressing genes in mouse outflow tract (HEM)**

Top 1,352 mouse genes that showed the highest expression in the OFT RNA-seq data in the wildtype embryos are listed. Genes were ranked according to the average fragments per kilobase per million reads (FPKM) calculated for each gene in the wildtype dataset.

**Supplemental Table 5.**  **Sequencing report of Bulk RNA-Sequencing performed by Ocean Ridge Biosciences**
