# Supplementary figures and images for: A Multi-Omics Approach Using a Mouse Model of Cardiac Malformations for Prioritization of Human Congenital Heart Disease Contributing Genes
Source: Front Cardiovasc Med. 2021 Aug 24;8:683074. doi: 10.3389/fcvm.2021.683074 (PMC8421733; doi:10.3389/fcvm.2021.683074)

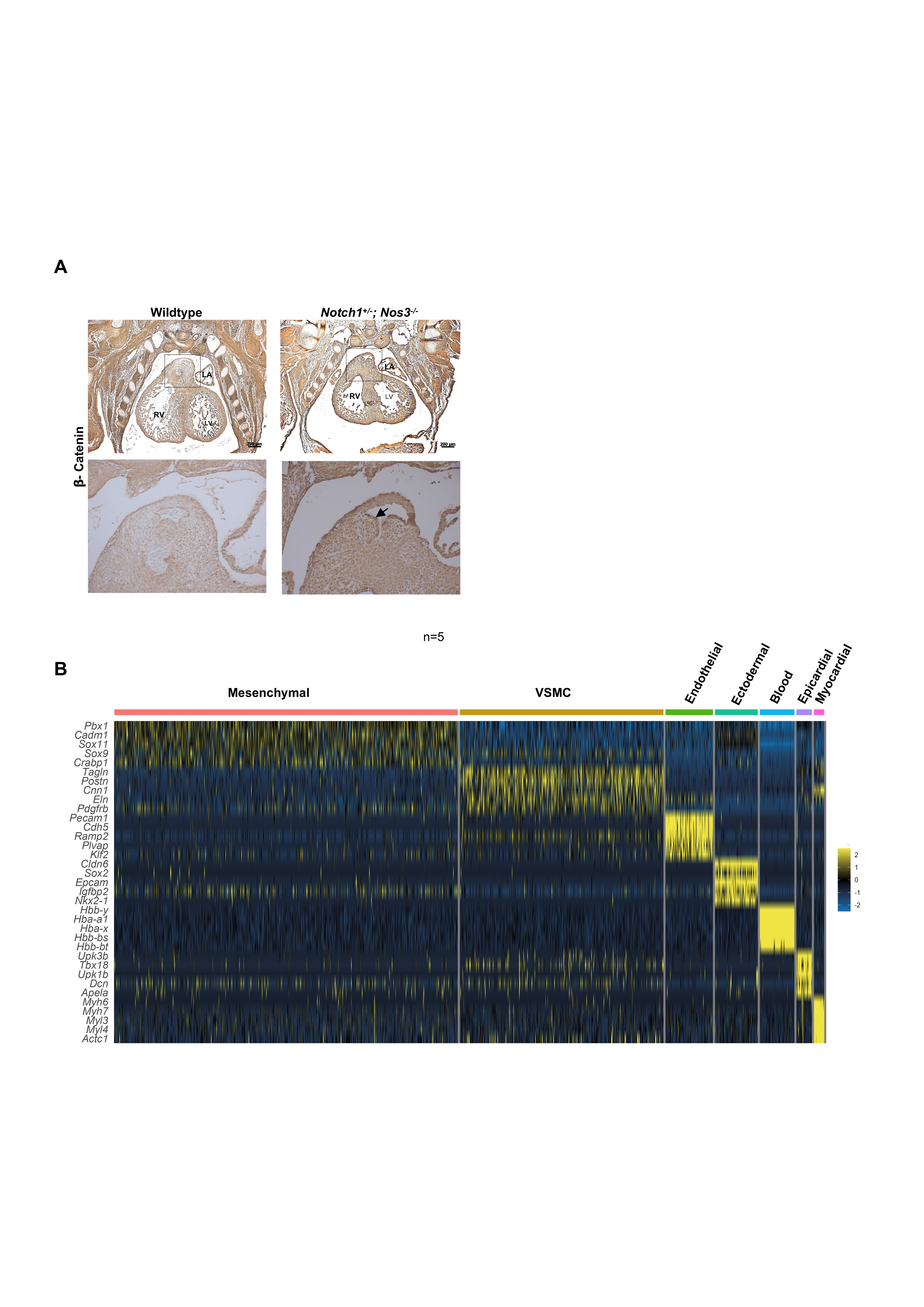

Supplement: Supplementary file 3 [file Image_1.JPEG]
